# Deep Learning-Based Image Quality Improvement in Digital Positron Emission Tomography for Breast Cancer

**DOI:** 10.3390/diagnostics13040794

**Published:** 2023-02-20

**Authors:** Mio Mori, Tomoyuki Fujioka, Mayumi Hara, Leona Katsuta, Yuka Yashima, Emi Yamaga, Ken Yamagiwa, Junichi Tsuchiya, Kumiko Hayashi, Yuichi Kumaki, Goshi Oda, Tsuyoshi Nakagawa, Iichiroh Onishi, Kazunori Kubota, Ukihide Tateishi

**Affiliations:** 1 Department of Diagnostic Radiology, Tokyo Medical and Dental University, 1-5-45 Yushima, Bunkyo-ku, Tokyo 113-8510, Japan; 2 Department of Surgery, Breast Surgery, Tokyo Medical and Dental University, 1-5-45 Yushima, Bunkyo-ku, Tokyo 113-8510, Japan; 3 Department of Comprehensive Pathology, Tokyo Medical and Dental University, 1-5-45 Yushima, Bunkyo-ku, Tokyo 113-8510, Japan; 4 Department of Radiology, Dokkyo Medical University Saitama Medical Center, 2-1-50 Minamiko-shigaya, Koshigaya 343-8555, Japan

**Keywords:** breast cancer, positron emission tomography, deep learning, image quality improvement

## Abstract

We investigated whether ^18^F-fluorodeoxyglucose positron emission tomography (PET)/computed tomography images restored via deep learning (DL) improved image quality and affected axillary lymph node (ALN) metastasis diagnosis in patients with breast cancer. Using a five-point scale, two readers compared the image quality of DL-PET and conventional PET (cPET) in 53 consecutive patients from September 2020 to October 2021. Visually analyzed ipsilateral ALNs were rated on a three-point scale. The standard uptake values SUV_max_ and SUV_peak_ were calculated for breast cancer regions of interest. For “depiction of primary lesion”, reader 2 scored DL-PET significantly higher than cPET. For “noise”, “clarity of mammary gland”, and “overall image quality”, both readers scored DL-PET significantly higher than cPET. The SUV_max_ and SUV_peak_ for primary lesions and normal breasts were significantly higher in DL-PET than in cPET (*p* < 0.001). Considering the ALN metastasis scores 1 and 2 as negative and 3 as positive, the McNemar test revealed no significant difference between cPET and DL-PET scores for either reader (*p =* 0.250, 0.625). DL-PET improved visual image quality for breast cancer compared with cPET. SUV_max_ and SUV_peak_ were significantly higher in DL-PET than in cPET. DL-PET and cPET exhibited comparable diagnostic abilities for ALN metastasis.

## 1. Introduction

^18^F-fluorodeoxyglucose positron emission tomography/computed tomography (^18^F-FDG PET/CT) is a noninvasive technique for detecting morphological changes and metabolic activities associated with malignancy [1,2]. It is useful for identifying distant metastases or secondary cancers associated with breast cancer and predicting their prognosis and therapeutic effects [1,3,4]. However, for several years, the noise and low spatial resolution of PET, which hinder its sensitivity, have been an issue [5,6,7]. Recently, PET scanners have been updated with technologies such as time-of-flight [2], digital output [8], and dedicated breast imaging [9] technologies to improve image quality.

Deep learning (DL) is a field of artificial intelligence wherein computers are not explicitly programmed but can analyze relationships among existing data and perform tasks based on new data [10]. DL has been applied in various fields, including breast PET-based research. Sato et al. created an Xception-based DL model using dedicated breast PET to detect breast cancer, reporting that its diagnostic performance was comparable with that of expert radiologists [11]. Takahashi et al. developed an Xception-based DL model that employed maximum-intensity projection PET images to detect breast cancer, revealing that its diagnostic performance was significantly better than that of the single radiologist participating in their study [12]. Moreau et al. used the no-new-Net method to segment bone and bone metastatic lesions of breast cancer [13]. Choi et al. used AlexNet to predict patient responses to neoadjuvant chemotherapy for advanced breast cancer. They found that compared with PET and magnetic resonance imaging (MRI) parameters, DL application improved the area under the curve [14].

DL technology is already in practical use to improve CT and MRI images by reducing noise and artifacts without reducing spatial resolution [15,16]. Replacing the image reconstruction process with DL improved the image quality of low-dose CT and shortened the MRI data acquisition time [15,16]. A previous study investigated the image quality improvement engendered by DL-based PET for 49 different malignancies and 1 case of Takayasu arteritis [17]. Another study evaluating the image quality obtained via digital PET/CT included 30 malignancies and 3 inflammations [18]. According to those studies, the noise was lower and lesion delineation and image quality were superior in the PET images restored using the DL method compared with the images produced using conventional reconstruction. Noise reduction using the DL algorithm increased the standard uptake values (SUVs) compared with those obtained via conventional reconstruction in most healthy tissues and tumors. However, the role of DL imaging in actual diagnosis is yet to be established [17,18].

Herein, we used the Advanced intelligent Clear-IQ Engine (AiCE), developed by Canon Medical Systems Corporation (Otawara, Japan) [18], to perform the DL method to investigate whether restoration via this method improved PET image quality and affected the diagnosis of ipsilateral axillary lymph node (ALN) metastasis in patients with breast cancer. To the best of our knowledge, this is the first study to examine the diagnostic ability of production-based PET using the DL algorithm with improved image quality. ^18^F-FDG PET/CT restored using the DL method might be useful for application in daily clinical practice.

## 2. Materials and Methods

### 2.1. Study Design and Patients

This retrospective study was approved by the medical ethics committee of our hospital (no. M2020-339).

The study enrolled consecutive patients with pathologically proven breast cancer who underwent ^18^F-FDG PET/CT before surgery or neoadjuvant therapy at our hospital from September 2020 to October 2021. Only the patients imaged using digital PET were enrolled. Two patients who had a recent history (within 2 months) of vaccination for COVID-19 on the shoulder ipsilateral to their breast cancer, which was likely to affect the diagnosis of ALN metastasis, were excluded. We excluded one patient with numerous superficial lymph nodes exhibiting FDG accumulation, probably owing to dermatitis. One patient with four simultaneous breast cancers was excluded as the breast cancer responsible for ALN accumulation could not be identified. One patient whose DL images were unavailable was excluded as the radiologist did not restore the DL images and deleted the raw data. The final cohort comprised 55 breast cancers in 53 patients. One patient had cancers in both the left and right breasts, and another patient had two cancers in the same breast.

### 2.2. ^18^F-FDG PET/CT Protocol

All patients were intravenously administered ^18^F-FDG (3.7 MBq/kg) after a minimum fasting period of 4 h. Whole-body images were routinely obtained using a digital PET/CT system (Cartesion Prime: Canon Medical Systems Corporation, Otawara, Tochigi, Japan). CT was performed using the following parameters: pitch, 0.813; gantry rotation time, 0.5 s; table time, 30 mm/s; autoexposure control (SD 20), 120 KVp; and slice thickness, 2.0 mm. Contrast materials were not used for the CT examinations. After ~60 min of ^18^F-FDG administration, whole-body emission PET was performed using the following parameters: emission time per bed, 90 s; bed position, 6–7; slice thickness, 2.11 mm; and matrix, 336 × 336.

### 2.3. Image Reconstruction

AiCE was used to obtain the DL-PET images. The conventional PET (cPET) images for comparison were reconstructed using the three-dimensional time-of-flight and ordered subset expectation maximization methods with 12 subsets, 2 iterations, scatter correction, attenuation correction, point spread function omission, and a Gaussian 3.00-mm postfilter.

### 2.4. Image Analysis

Two breast radiologists with 8 and 13 years of experience in breast imaging individually used EV Insite R viewer software (PSP Corporation, Tokyo, Japan) to visually assess the axial PET images. The radiologists were blinded to whether they were assessing DL-PET or cPET images. They were aware of the position of the breast cancers in the left and right breast quadrants. The “depiction of primary lesion” score was defined as either 1, not visible; 2, indistinguishable from the background; 3, slight accumulation; 4, clearly visible; or 5, very clearly visible. The “noise” score was defined as either 1, not suitable for diagnosis; 2, noise inseparable from the accumulation; 3, noise that did not obscure the accumulation; 4, little noise; or 5, almost no noise. “Clarity of mammary gland” and “overall image quality” scores were both defined as either 1, extremely poor; 2, poor; 3, fair; 4, good; or 5, excellent.

Both radiologists further visually analyzed the ipsilateral ALNs, which they scored as either 1, negative; 2, intermediate; or 3, positive, with 1 indicating no evident ALN metastasis and 3 indicating the presence of ALN metastasis. If ALN accumulation was clear but the presence or absence of metastasis was difficult to determine, a score of 2 was given. For one patient with duplicate unilateral breast cancers, we considered that the ALN metastasis was associated with the larger breast cancer.

A region of interest was circled on each breast cancer image to calculate the SUV_max_ and SUV_peak_ by a reader with 8 years of experience in breast imaging using a VOX-BASE image reader (version 2.8: J-Mac System, Inc., Hokkaido, Japan). The region of interest located on the contralateral breast at the maximum accumulation site, excluding the nipple, was considered the normal breast reference. A patient whose data was not sent to VOX-BASE was excluded. The contralateral breast SUV of one patient with ipsilateral double breast cancer was measured for the larger breast cancer. For one patient with bilateral breast cancer, the region of interest was selected to exclude the breast cancers to evaluate the normal breast.

### 2.5. Clinicopathologic Evaluation

Age, sex, and pathology data were obtained from the medical records of the patients. The surgical specimens of the breast tissue were sectioned by pathologists into 5–10 mm contiguous sections, and thinner slices were obtained as required. All surgical and biopsy specimens were evaluated by a pathologist who recorded the following histologic features: tumor size of an invasive component; nuclear grade (1, 2, or 3); presence of vascular and lymphatic invasion; receptor status (estrogen receptor, progesterone receptor, or HER2); and Ki-67 staining. A cutoff of 1% was used for the estrogen and progesterone receptor assays, as specified in the American Society of Clinical Oncology/College of American Pathologists guidelines [19]. The HER2 status was considered positive for 3+ scores in immunohistochemistry or if fluorescence in situ hybridization demonstrated gene amplification [20].

### 2.6. Statistical Analysis

Any difference in each visual analysis score between cPET and DL-PET was evaluated using the IBM SPSS Statistics software application (version 24: IBM Corporation, NY, USA). Kolmogorov–Smirnov test was used to assess whether SUVs were normally distributed. Two-tailed paired samples *t*-test was used for normal distributions and Wilcoxon signed-rank test was used for non-normal distributions to confirm whether SUVs differed significantly between the DL-PET and cPET images. Marginal homogeneity and McNemar tests were performed to investigate whether the ALN metastasis scores of the cPET and DL-PET matched. A *p*-value of <0.05 was considered statistically significant.

## 3. Results

### 3.1. Patients

All 53 patients with 55 breast cancers were women. The mean age of the cohort was 59.85 ± 14.38 (range, 37–88) years. Of the 53 patients, 51 underwent biopsy at an average of 25.35 ± 20.88 (range, 1–41) days before ^18^F-FDG PET/CT. Of the 45 patients who underwent surgery, the mean interval from ^18^F-FDG PET/CT to surgery was 37.51 ± 15.54 (range, 9–76) days. Surgery revealed invasive ductal carcinomas of no special type (*n* = 34) and in situ ductal (*n* = 6), invasive lobular (*n* = 1), mucinous (*n* = 1), apocrine (*n* = 1), secretory (*n* = 1), invasive solid papillary (*n* = 1), micro-invasive ductal (*n* = 1), and micro-invasive apocrine (*n* = 1) carcinomas. The average invasive diameter of the resected breast cancers was 14.10 ± 10.83 (range, 0–40) mm. The remaining 8 lesions were diagnosed as invasive ductal carcinomas of no special type (*n* = 7) or invasive lobular carcinoma (*n* = 1) via biopsy. The average Ki-67 staining in the 55 breast cancers was 23.52% ± 15.89% (range, 1–63.9%). Table 1 presents the biomarker status of the cancers.

### 3.2. Visual Analysis

**Figure 1:**
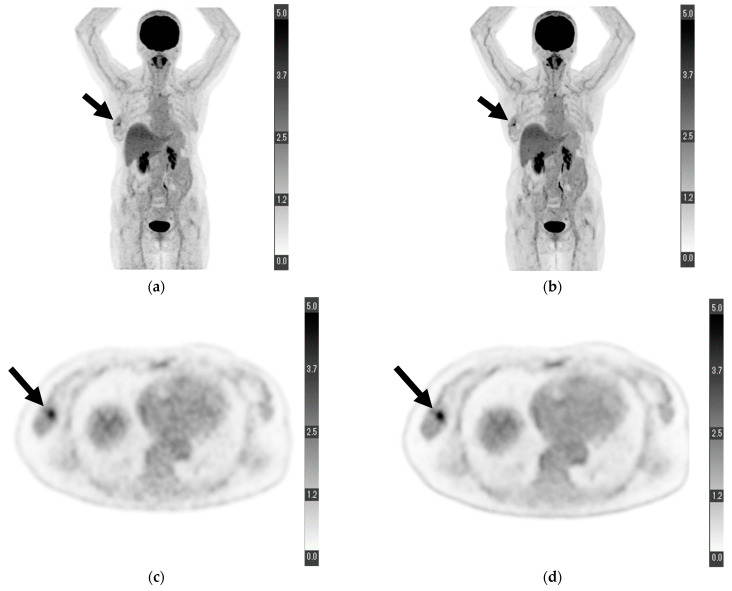
Maximum-intensity projection of (**a**) conventional positron emission tomography (PET) reconstruction and (**b**) deep learning (DL) PET restoration of a woman in her 60 s with invasive ductal carcinoma in the right breast (arrows): invasive diameter, 15 mm; nuclear grade, 2; lymphatic invasion, negative; vascular invasion, negative; estrogen receptor, positive; progesterone receptor, positive; HER2 receptor, positive; Ki-67 staining, 25.5%; and nodal stage, 0. Axial view of a tumor cross-section for the same woman using (**c**) cPET reconstruction and (**d**) DL-PET restoration. Images were scored on a five-point scale from 1 = extremely poor to 5 = excellent. The scores of reader 1 for “depiction of the primary lesion”, “noise”, “clarity of mammary gland”, and “overall image quality” were 4, 5, 5, and 5 for cPET and 5, 5, 5, and 5 for DL-PET, respectively; the scores of reader 2 were 3, 4, 4, and 4 for cPET and 5, 5, 5, and 5 for DL-PET, respectively.

For “depiction of primary lesion”, the average score provided by reader 1 was higher for DL-PET than for cPET; however, no significant difference was observed (4.673 ± 0.695 vs. 4.455 ± 0.857, *p =* 0.121). Conversely, the score provided by reader 2 for DL-PET was significantly higher than that for cPET (4.364 ± 0.969 vs. 4.000 ± 1.089, *p =* 0.049). The scores of readers 1 and 2 for DL-PET were significantly higher than those for cPET in terms of “noise” (reader 1, 4.782 ± 0.498 vs. 4.073 ± 0.663, *p* < 0.001; reader 2, 4.491 ± 0.573 vs. 3.855 ± 0.621, *p* < 0.001), “clarity of mammary gland” (reader 1, 4.309 ± 0.836 vs. 3.909 ± 0.727, *p =* 0.003; reader 2, 4.109 ± 0.737 vs. 3.291 ± 0.737, *p* < 0.001), and “overall image quality” (reader 1, 4.636 ± 0.557 vs. 4.073 ± 0.663, *p* < 0.001; reader 2, 4.473 ± 0.539 vs. 3.904 ± 0.470, *p* < 0.001). The relevant data are shown in Table 2. Figure 1 shows a sample PET from a patient with some visual analysis scores higher in DL-PET than in cPET.

### 3.3. SUVs

Table 3 presents the results of the Kolmogorov–Smirnov test, and Table 4 presents the results of the two-tailed paired samples *t*-test and Wilcoxon signed-rank test. The SUV_max_ and SUV_peak_ in primary breast cancer were significantly higher in DL-PET restorations than in cPET reconstructions (SUV_max_, 8.954 ± 6.544 vs. 7.220 ± 5.161, *p* < 0.001; SUV_peak_, 4.564 ± 3.828 vs. 4.413 ± 3.667, *p* < 0.001). Similarly, in normal breast tissue, the SUV_max_ and SUV_peak_ were observed to be significantly higher in DL-PET restorations than in cPET reconstructions (SUV_max_, 1.771 ± 0.716 vs. 1.612 ± 0.572, *p* < 0.001; SUV_peak_, 1.612 ± 0.572 vs. 1.235 ± 0.393, *p* < 0.001).

### 3.4. ALN Metastasis

The group positive for ALN metastases included 13 breast cancers diagnosed by surgery or biopsy in 13 patients and 1 stage IV cancer in a patient with clearly imaged metastases. The group negative for ALN metastasis included 33 patients with no metastases on surgery or biopsy and 3 patients with metastases <2 mm (N1 mi). Three patients whose ALNs were not pathologically diagnosed or who had smaller unilaterally duplicated breast cancers were excluded.

**Figure 2:**
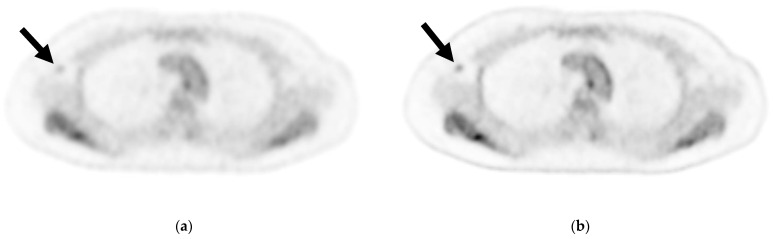
Axial image of an axillary lymph node (ALN) cross-section via (**a**) cPET and (**b**) DL-PET of a woman in her 40 s with invasive lobular carcinoma in the right breast: invasive diameter, 18 mm; nuclear grade, 1; lymphatic invasion, positive; vascular invasion, positive; estrogen receptor, positive; progesterone receptor, positive; HER2 receptor, negative; Ki-67 staining, 17.3%; and nodal stage, 1a. Of 18 ALNs, 3 were positive for metastasis; the maximum diameter was 3 mm, and an extranodal invasion was evident. Visual analysis for ipsilateral ALN metastasis was rated on a scale of 1–3 (1 = negative, 2 = intermediate, 3 = positive). For ALN metastasis, reader 1 gave a score of 2 for cPET and 3 for DL-PET, and reader 2 gave a score of 1 for both the PET types. cPET, PET with conventional reconstruction; DL-PET, PET with deep learning restoration.

Table 5 shows the results of the visual analysis for the presence or absence of ALN metastasis. The marginal homogeneity test revealed a significant difference in the scores assigned to cPET reconstructions and DL-PET restorations by reader 1 (*p =* 0.034) but not by reader 2 (*p =* 0.439). When the scores of 1 and 2 were defined as negative and a score of 3 was defined as positive, the sensitivity, specificity, positive predictive value, negative predictive value, and accuracy were 57.14%, 94.59%, 80.00%, 85.37%, and 84.31% for cPET and 64.29%, 89.19%, 69.23%, 86.84%, and 82.35% for DL-PET, respectively, according to reader 1. Per the McNemar test, the scores of reader 1 for cPET and DL-PET were not significantly different (*p =* 0.250). According to the evaluation of reader 2, the sensitivity, specificity, positive predictive value, negative predictive value, and accuracy were 42.86%, 94.59%, 75.00%, 81.40%, and 80.39% for cPET and 50.00%, 91.89%, 70.00%, 82.93%, and 80.39% for DL-PET, respectively (Table 6). Per the McNemar test, the scores of reader 2 for cPET and DL-PET were also not significantly different (*p =* 0.625). Figure 2 shows a sample axial PET image of a patient with ALN metastasis.

## 4. Discussion

Herein, DL-PET improved the visualization of primary breast cancer and mammary gland contrast, reduced visual noise, and significantly increased SUV_max_ and SUV_peak_, suggesting that new criteria should be established for interpreting DL-PET. DL-PET exhibits an efficiency equivalent to that of cPET for ALN diagnosis.

Currently, DL has been applied to various fields, including breast PET-based research. There have been studies regarding breast cancer classification according to dedicated breast [11] and multiple-intensity projection of whole-body PET [12], bone lesion segmentation [13], and neoadjuvant chemotherapy response prediction using PET and MRI [14]. However, few studies involving PET have used DL to improve image quality, and to the best of our knowledge, there have been no reports regarding its application in breast-related diseases. There have been studies with the aim of improving PET image quality from the perspective of attenuation correction, which have generated attenuation maps [21] and predicted patient-specific attenuation correction factors [22] using convolution neural networks and paired attenuation- and non-attenuation-corrected PET images employing three-dimensional patch-based cycle-consistent generative adversarial networks [23]. A study by Park et al. involving the PET/MRI of 20 patients with Crohn’s disease reported that the image quality of low-dose PET with 90% dose reduction was potentially comparable with that of full-dose PET using the DL technique [24].

Herein, image quality was significantly improved in DL-PET than in cPET, consistent with the results of previous studies [13,14], and restored the image quality preferred by readers. Numerous studies have analyzed the relationship between ^18^F-FDG uptake and pathological findings of breast cancer to predict disease prognosis and chemotherapy efficacy [1,3,25,26]. The noise reduction and improved lesion/normal tissue contrast achieved with DL-PET might make a more accurate prediction of chemotherapy efficacy. Furthermore, the improved image quality of PET/CT can be achieved with tracers other than ^18^F-FDG, such as ^18^F-FLT [27]. The difference between the conventional and DL approaches explains the significantly higher SUV_max_ and SUV_peak_ obtained in DL-PET than in cPET. Gaussian filtering shaves off pixel values in the images, whereas DL only removes noise [13,14]. A meta-analysis revealed that patients with breast cancer and high SUV_max_ were at an increased risk of adverse events or death, with high metabolic tumor volume and total lesion glycolysis predicting a high risk of death and adverse events, respectively [4]. When using DL-PET to predict tumor activity and chemotherapy efficacy, it is necessary to understand that SUVs will be higher for DL-PET than for cPET.

Although the contrast between the background axillary adipose tissue and ALNs improved, it did not improve the diagnosis of ALN metastases while using DL-PET (Figure 2). The DL-PET images in metastasis-negative cases were scored 2 or 3 by reader 1 more frequently compared with cPET images. It was difficult to determine whether the ^18^F-FDG accumulation on DL-PET was associated with metastasis because the accumulation was clear regardless of the presence or absence of ALN metastasis. When scores of 1 and 2 were defined as negative and a score of 3 as positive, both readers reached the same visual diagnosis with cPET reconstructions and DL-PET restorations. When evaluating high-contrast images, such as those obtained using DL-PET, moderate accumulation requires careful assessment to ensure it is not mistakenly labeled as positive for metastasis. In this study, cPET, which was considered as the baseline, was performed using the latest digital PET scanner technology. Hence, it is possible that the image quality was too good to demonstrate a significant difference between cPET and DL-PET.

Future studies with larger sample sizes are warranted to confirm that lowering the ^18^F-FDG dose and shortening the emission time per bed do not compromise the visual diagnosis of ALN metastases owing to the improvement in the quality of PET images obtained via DL restoration. The patient benefits of using the DL method might include low testing costs and exposure doses by lowering examination time and the ^18^F-FDG dose [24].

Our study has some limitations. First, this was a retrospective single-center study. Second, it included a relatively small sample size. Third, we did not evaluate the diagnostic abilities of DL-PET and cPET for distant metastasis apart from ALNs, as most patients had early-stage breast cancer. Furthermore, no SUV_mean_ measurements or SUV histograms were obtained because breast cancer is often irregular and ^18^F-FDG uptake is very low, rendering it inseparable from the background mammary gland and making it difficult to determine accurate margins.

In conclusion, compared with cPET, DL-PET improved the quality of the resulting breast cancer images. The SUV_max_ and SUV_peak_ were significantly higher in DL-PET than in cPET reconstructions owing to selective noise reduction. Thus, DL-PET and cPET exhibited comparable abilities to diagnose ALN metastasis. Therefore, the DL method has the potential to generate high-quality PET images while preserving the ability to visually diagnose ALNs.

## Figures and Tables

**Table 1:** Pathology results of the 55 breast cancers.

Variable	Status	Result
Nuclear grade *	1	28
	2	17
	3	8
Lymphatic invasion **	Positive	13
	Negative	34
Vascular invasion **	Positive	12
	Negative	35
Estrogen receptor	Positive	48
	Negative	7
Progesterone receptor	Positive	46
	Negative	9
HER2	Positive	16
	Negative	39

HER2, human epidermal growth factor receptor 2. * Data for two breast cancers lost. ** Limited to 47 resected breast cancers.

**Table 2:** Visual analysis scores.

	Reader 1			Reader 2		
	Score *		*p* Value **	Score *		*p* Value **
	Conventional	DL		Conventional	DL	
Depiction of primary lesion	4.455 ± 0.857	4.673 ± 0.695	0.121	4.000 ± 1.089	4.364 ± 0.969	**0.049**
Noise	4.073 ± 0.663	4.782 ± 0.498	<0.001	3.855 ± 0.621	4.491 ± 0.573	**<0.001**
Clarity of mammary gland	3.909 ± 0.727	4.309 ± 0.836	0.003	3.291 ± 0.737	4.109 ± 0.737	**<0.001**
Overall image quality	4.073 ± 0.663	4.636 ± 0.557	<0.001	3.904 ± 0.470	4.473 ± 0.539	**<0.001**

* Evaluated on a five-point scale (1 = extremely poor, 2 = poor, 3 = fair, 4 = good, and 5 = excellent); numbers in the table show the average score ± standard deviation. ** Significant values are shown in boldface type. Conventional, positron emission tomography (PET) with conventional reconstruction; DL, PET with deep learning restoration.

**Table 3:** Kolmogorov–Smirnov test for standardized uptake values (SUVs).

Tissue Type	Reconstruction Type	*p* Value *
SUV_max_ for primary lesions	Conventional	**0.001**
	DL	**<0.001**
SUV_peak_ for primary lesions	Conventional	**<0.001**
	DL	**<0.001**
SUV_max_ for normal breast tissue	Conventional	0.200
	DL	**0.040**
SUV_peak_ for normal breast tissue	Conventional	0.200
	DL	0.164

* Significant values are shown in boldface type. Wilcoxon signed-rank test was used for non-normally distributed variables. Conventional, PET with conventional reconstruction; DL, PET with deep learning restoration.

**Table 4:** SUVs.

Reconstruction Type	Primary Lesion		Normal Breast	
	SUV_max_	SUV_peak_	SUV_max_	SUV_peak_
Conventional	7.220 ± 5.161	4.413 ± 3.667	1.612 ± 0.572	1.235 ± 0.393
DL	8.954 ± 6.544	4.564 ± 3.828	1.771 ± 0.716	1.276 ± 0.415
*p*-value *	**<0.001**	**<0.001**	**<0.001**	**<0.001**

* Significant values are shown in boldface type. Wilcoxon signed-rank test was used for the SUV_max_ and SUV_peak_ of primary lesions and the SUV_max_ of normal breast tissue. Two-tailed paired samples *t*-test was used for the SUV_peak_ of normal breast tissue. Conventional, PET with conventional reconstruction; DL, PET with deep learning restoration.

**Table 5:** Scores by each reader for axillary lymph node (ALN) metastasis on visual analysis *.

ALN Metastasis	PET Type	Reader 1 Scoring			Reader 2 Scoring		
		1	2	3	1	2	3
Positive	Conventional	1	5	8	4	4	6
	DL	2	3	9	5	2	7
Negative	Conventional	27	8	2	30	5	2
	DL	23	10	4	28	6	3

* Rated on a scale of 1–3 (1 = negative, 2 = intermediate, and 3 = positive). The numbers in the table indicate the number of breast cancers assigned to the indicated score. Positive, the group of patients positive for ALN metastasis (*n* = 14); Negative, the group of patients negative for ALN metastasis (*n* = 37); Conventional, PET with conventional reconstruction; DL, PET with deep learning restoration.

**Table 6:** Diagnostic accuracy of ALN metastasis *.

		Sensitivity (%)	Specificity (%)	PPV (%)	NPV (%)	Accuracy (%)
Reader 1	Conventional	57.14	94.59	80.00	85. 37	84.31
	DL	64.29	89.19	69.23	86.84	82.35
Reader 2	Conventional	42.86	94.59	75.00	81.40	80.39
	DL	50.00	91.89	70.00	82.93	80.39

* When scores of 1 and 2 were defined as negative and a score of 3 was defined as positive. PPV, positive predictive value; NPV, negative predictive value; Conventional, PET with conventional reconstruction; DL, PET with deep learning restoration.

## Data Availability

The data presented in this study are available on request from the corresponding author. The data are not publicly available due to protect patient anonymity.
